# Supplementary material for: Towards unified AI-driven fracture mechanics: the extended deep energy method (XDEM)
Source: Nat Commun. 2026 Aug 15;17:8492. doi: 10.1038/s41467-026-76748-1 (PMC13484471; doi:10.1038/s41467-026-76748-1)
Supplement: Supplementary file 3 [file 41467_2026_76748_MOESM3_ESM.zip › pic/Results/Crack_propagation/Straight/3D_fdGraph.pdf]

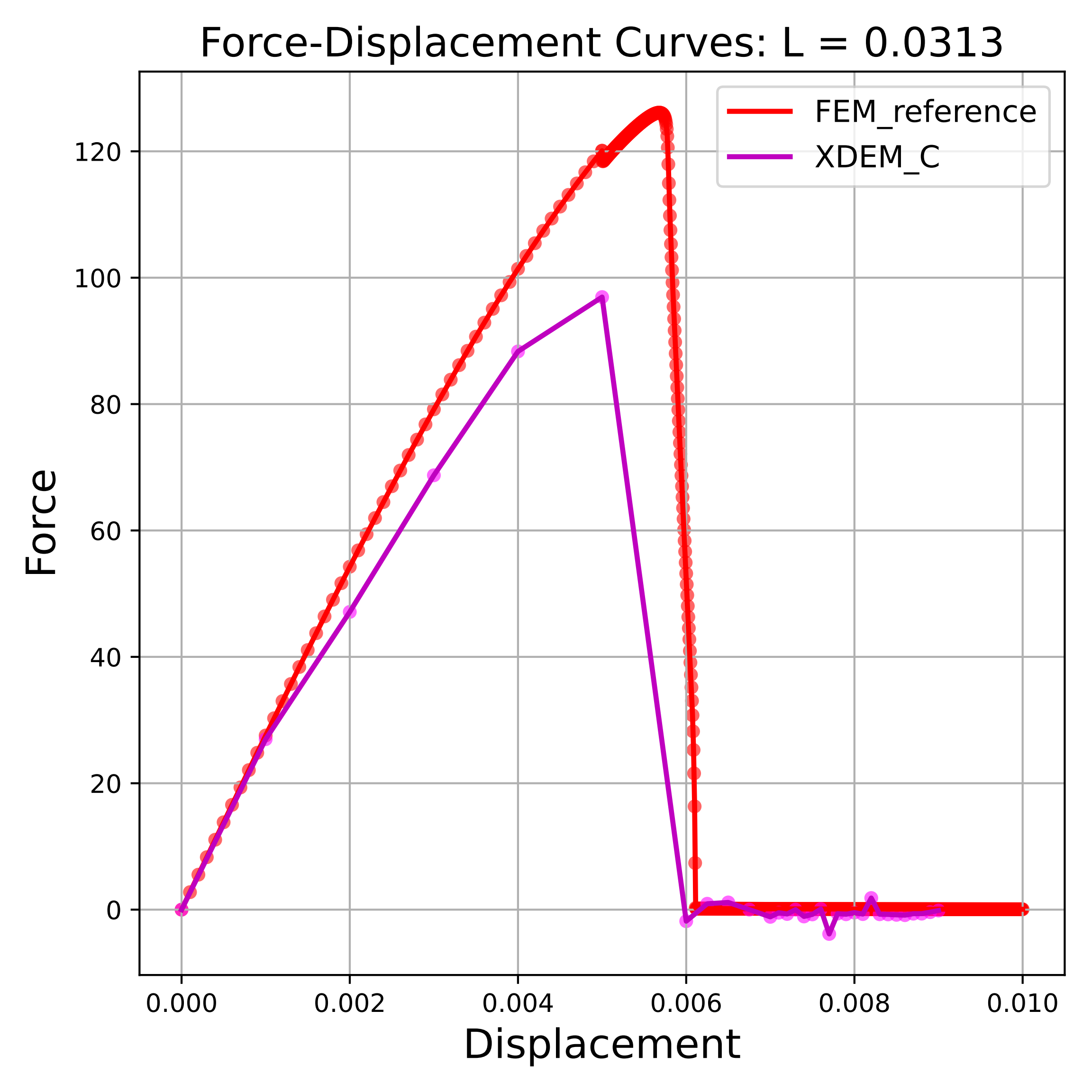
Force-Displacement Curves: L = 0.0313
FEM_reference
XDEM_C
Force
Displacement
0
20
40
60
80
100
120
0.000
0.002
0.004
0.006
0.008
0.010
